# Research on interventional treatment strategies for lower extremity deep venous thrombosis based on real-world data

**DOI:** 10.3389/fcvm.2026.1793706

**Published:** 2026-05-04

**Authors:** Guili Wang, Lifeng Qu, Nuan Wen, Donglin Lu, Xiwen Liu, Zhaoxuan Liu

**Affiliations:** 1 Department of Vascular Surgery, Central Hospital Affiliated to Shandong First Medical University, Jinan, China; 2 Medical Imaging Center - Interventional Department, Central Hospital Affiliated to Shandong First Medical University, Jinan, China

**Keywords:** Angiojet ®, deep vein thrombosis, endovascular treatment, mechanical thrombectomy, treatment strategy

## Abstract

**Objective:**

Current treatment strategies for acute lower extremity deep vein thrombosis (DVT) have shifted from merely preventing pulmonary embolism to actively removing thrombus and preserving valve function. However, there is limited evidence comparing the indications and perioperative outcomes of different endovascular strategies. This study aims to investigate the differences in indications, perioperative characteristics, and efficacy trends among three strategies: Angiojet mechanical thrombectomy alone, other thrombus removal devices alone, and a combination of Angiojet with other devices.

**Methods:**

A retrospective analysis was conducted on the clinical data of 232 DVT patients who underwent endovascular treatment in the Vascular Surgery Department of our hospital between January 2022 and April 2025. Based on the core thrombus removal method, patients were divided into three groups: Group A (Angiojet alone, *n* = 22), Group B (other devices alone, including manual aspiration and catheter-directed thrombolysis CDT, *n* = 35), and Group C (Angiojet combined with other devices, *n* = 175). Baseline characteristics, thrombus anatomical extent, and perioperative parameters were compared across the groups. Chi-square test, analysis of variance, or Kruskal–Wallis test were used for intergroup comparisons.

**Results:**

There were fundamental differences in thrombus burden among the three groups (*P* < 0.001). Groups A and B primarily treated limited (femoral/popliteal) thrombosis (50.0% and 51.4%, respectively), while Group C was the predominant approach for extensive thrombosis (iliofemoral-popliteal, 50.9%) and thrombosis involving the inferior vena cava (22.3%). The operative time for Group C (134 ± 48 min) was significantly longer than for Group A (98 ± 36 min) and Group B (115 ± 45 min) (*P* < 0.001), and blood loss (66 ± 39 mL) was also higher compared to the other two groups (*P* = 0.008). In terms of treatment intensity, the balloon angioplasty rate (96.0%) and stent implantation rate (58.9%) in Group C were significantly higher than in Groups A and B (*P* < 0.001). Group B showed the highest trend for documented “partial thrombus residue” (22.9%).

**Conclusion:**

Current endovascular DVT treatment has formed a clear risk-stratified decision-making pathway. Angiojet alone is an efficient and streamlined option for limited acute DVT; for extensive and complex DVT, the intensified strategy of Angiojet combined with other thrombus removal devices has become the standard choice, with its longer operative time and higher rate of vascular interventions reflecting disease complexity. This study provides real-world evidence for individualized endovascular DVT treatment.

## 1 Introduction

Acute lower extremity deep vein thrombosis (DVT) is a common vascular emergency. The treatment goals have evolved from preventing pulmonary embolism to actively removing thrombus, preserving valve function, and preventing post-thrombotic syndrome (PTS) (1, 2). Currently, the therapeutic armamentarium for DVT includes anticoagulation alone, catheter-directed thrombolysis (CDT), percutaneous mechanical thrombectomy (PMT), and surgical thrombectomy. Anticoagulation remains the cornerstone for preventing thrombus extension and recurrence but does not actively remove existing clot. CDT was once the mainstay but is limited by high bleeding risk and prolonged hospital stay (3, 4).

In recent years, PMT has been widely adopted for its ability to rapidly reduce thrombus burden and decrease thrombolytic drug dosage (5, 6). Among these, the Angiojet Thrombectomy System (Boston Scientific, USA), a hydrodynamic PMT device that utilizes a combination of thrombolytic drug injection and Bernoulli-effect aspiration, holds a significant position in clinical practice (7, 8). Surgical thrombectomy is reserved for rare cases with contraindications to endovascular approaches or failed catheter-based interventions. Among these, endovascular strategies have gained prominence due to their minimally invasive nature and versatility in tailoring treatment to thrombus burden and anatomical complexity. However, facing thrombi of varying durations and anatomical extents, a single device often proves insufficient. In clinical practice, vascular surgeons frequently tailor treatment plans by flexibly combining various devices such as the Angiojet system, guiding catheter manual aspiration, and catheter-directed thrombolysis, forming personalized therapeutic regimens (9, 10).

Currently, there is a lack of studies systematically comparing the suitable patient populations and clinical outcomes of three common strategies: Angiojet alone, other thrombus removal devices alone, and the combination of Angiojet with other devices. Clarifying the clinical decision-making logic and perioperative characteristics of different strategies is crucial for optimizing treatment pathways and achieving precision medicine (11, 12). This study aims to analyze the differences in patient selection, surgical features, and efficacy trends among three endovascular treatment strategies using single-center, large-sample, real-world data, thereby providing evidence for clinical decision-making.

## 2 Methods

### 2.1 Study design and patient population

This single-center retrospective observational study was granted an exemption from review and informed consent by the hospital's Ethics Committee. Consecutive patients who underwent endovascular thrombus removal treatment for acute or subacute symptomatic lower extremity DVT in our Vascular Surgery Department between January 2022 and April 2025 were included. Inclusion criteria: 1) Acute (symptom duration ≤14 days) lower extremity DVT diagnosed by venous ultrasound or CT venography; 2) First-time endovascular thrombus removal treatment; 3) Complete clinical data. Exclusion criteria: 1) Anticoagulation or systemic thrombolysis only; 2) Chronic total occlusive thrombus; 3) Concurrent active bleeding or severe coagulation dysfunction.

### 2.2 Group definition

Patients were divided into three groups based on the core thrombus removal method documented in the operative notes:

#### 2.1.1 Group A (Angiojet alone)

The Angiojet system (6F or 8F) was used as the sole device for thrombus removal, without combination with other aspiration or thrombolytic devices.

#### 2.1.2 Group B (other devices alone, including manual aspiration and CDT)

The Angiojet system was not used. Thrombus removal relied solely on other devices, mainly including: manual aspiration using large-bore (e.g., ≥8F) guiding catheters or sheaths, aspiration using intermediate catheters (e.g., 6F Neuron MAX), and catheter-directed thrombolysis.

When CDT was performed, urokinase was infused at a dose of 200,000–500,000 IU over 12–24 h through a multi-side-hole Fountain catheter positioned within the thrombus.

#### 2.1.3 Group C (Angiojet combined)

The Angiojet system was used in combination with other thrombus removal devices (e.g., large-bore guiding catheters, sheaths) for thrombus clearance.

In Groups A and C, the Angiojet system was used, exclusively with Solent series catheters (including Omni and Proxi variants). No AVX system was employed. The 8F catheter was preferred for iliac and extensive thrombi, while the 6F catheter was selected for femoropopliteal involvement. The CDT technique was consistent across Groups B and C, as described above.

### 2.3 Data collection

The following data were collected via the electronic medical record system: 1) Baseline data: Age, sex, height, weight, smoking history, comorbidities (hypertension, diabetes, coronary artery disease, malignancy, etc.); 2) Thrombus characteristics: affected limb, symptom duration (days of swelling), and thrombus anatomical extent (categorized by imaging: limited, femoral/popliteal veins; extensive, iliofemoral-popliteal veins; central, involvement of the inferior vena cava and/or iliac/femoral veins); 3) Surgical details: Access site, Angiojet model used, types of other devices used in combination, whether catheter-directed thrombolysis was performed, whether balloon angioplasty and stent implantation were performed, and stent model/quantity; 4) Perioperative parameters included operative time (from puncture to dressing completion), intraoperative blood loss, length of hospital stay, and documentation of “partial thrombus residue.” This finding was defined as visible residual thrombus occupying ≤30% of the venous lumen on intraoperative venography, as recorded in the operative note by the operating surgeon.

### 2.4 Statistical analysis

Statistical analysis was performed using SPSS software (version 26.0). Normally distributed continuous variables were expressed as mean ± standard deviation (x¯ ± s) and compared using one-way ANOVA. Non-normally distributed continuous variables were expressed as median (interquartile range) [M (IQR)] and compared using the Kruskal–Wallis *H* test. Categorical variables were expressed as number (percentage) [n (%)] and compared using the Chi-square test or Fisher's exact test. A *P*-value <0.05 was considered statistically significant.

## 3 Results

### 3.1 Patient baseline characteristics

A total of 232 patients were included in the analysis: 22 in Group A, 35 in Group B, and 175 in Group C. There were no statistically significant differences among the three groups in age, sex, or common comorbidities (hypertension, diabetes, coronary artery disease) (*P* > 0.05), although certain trends were observed (Table 1). Group B had the highest mean age (72.1 ± 10.8 years) and the highest proportion of patients with a history of malignancy (25.7%), though this did not reach statistical significance (*P* = 0.184). All three groups predominantly involved the left limb (overall 70.7%), with no intergroup difference.

**Table 1:** Baseline demographic of patients in the three treatment groups.

Characteristic	Overall (*N* = 232)	Group A: Angiojet Alone (*n* = 22)	Group B: Other Devices Alone (*n* = 35)	Group C: Angiojet Combined (*n* = 175)	*P*-value
**Demographics**					
Age, years, mean ± SD	69.2 ± 11.5	66.8 ± 13.1	72.1 ± 10.8	69.0 ± 11.4	0.062
Female, *n* (%)	141 (60.8)	13 (59.1)	20 (57.1)	108 (61.7)	0.892
**Comorbidities, *n* (%)**					
Hypertension	138 (59.5)	11 (50.0)	20 (57.1)	107 (61.1)	0.598
Diabetes Mellitus	57 (24.6)	4 (18.2)	10 (28.6)	43 (24.6)	0.637
Coronary Artery Disease	52 (22.4)	4 (18.2)	7 (20.0)	41 (23.4)	0.826
History of Malignancy	38 (16.4)	2 (9.1)	9 (25.7)	27 (15.4)	0.184
**Limb Involvement**					
Left Limb, *n* (%)	164 (70.7)	14 (63.6)	24 (68.6)	126 (72.0)	0.668

SD, Standard Deviation.

### 3.2 Thrombus characteristics and strategy selection

**Figure 1:**
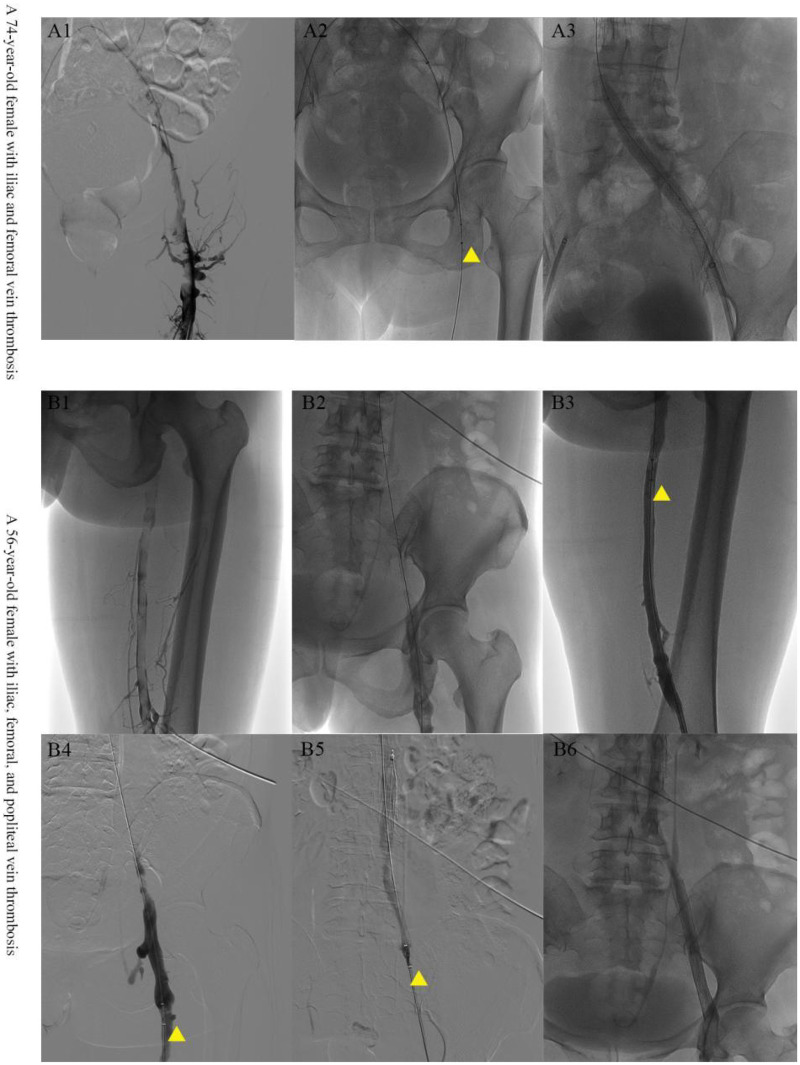
Deep vein thrombosis in two patients (A1-A3, B1-B6) was managed primarily with the angiojet thrombectomy device, supplemented by balloon angioplasty and venous stenting. Yellow arrows denote the working segment of the Angiojet catheter.

Thrombus anatomical extent was the most significant difference among the three groups (*P* < 0.001, Table 2). Groups A and B primarily treated limited (femoral/popliteal) thrombosis, with proportions of 50.0% and 51.4%, respectively. In contrast, Group C was the predominant strategy for extensive thrombosis: it handled “iliofemoral-popliteal” extensive thrombosis in 50.9% of cases and thrombosis involving the “inferior vena cava” in a significantly higher proportion (22.3%) compared to Group A (0%) and Group B (5.7%). As shown in Figure 1, two representative cases of limited DVT successfully achieved thrombus clearance using the Angiojet device, followed by venous flow restoration with adjunctive balloon angioplasty and stenting. This visual evidence corroborates the clinical application of the Group A strategy primarily for limited thrombus burden. Regarding symptom duration, Group B had the longest median duration of limb swelling [5 (2–10) days], suggesting this group may have included more subacute or chronic thrombi.

**Table 2:** Thrombus anatomical distribution and symptom duration across treatment groups.

Characteristic	Overall (*N* = 232)	Group A (*n* = 22)	Group B (*n* = 35)	Group C (*n* = 175)	*P*-value
**Thrombus Anatomical Extent, *n* (%)**					
Limited (Femoral/Popliteal)	76 (32.8)	11 (50.0)	18 (51.4)	47 (26.9)	**<0**.**001**
Extensive (Iliofemoral-Popliteal)	115 (49.6)	11 (50.0)	15 (42.9)	89 (50.9)	
Involving Inferior Vena Cava (IVC)	41 (17.7)	0 (0)	2 (5.7)	39 (22.3)	
**Symptom Duration**					
Days of Swelling, median (IQR)	4 (2, 7)	3 (1, 5)	5 (2, 10)	4 (2, 7)	0.051

IVC, Inferior Vena Cava; IQR, Interquartile Range.

Bold *P*-value <0.05 indicates statistical significance.

### 3.3 Surgical details and device application

The choice of access correlated with thrombus location. Group C, dealing more with iliac vein pathology, had a higher proportion of cases utilizing “crossover” access (39.4%) compared to Group A (18.2%). In both Groups A and C, the 8F Angiojet was the primary model used (72.7% and 69.7%, respectively). The core technique in Group B was manual aspiration using large-bore guiding catheters (100%). Regarding combination strategy, in Group C, over 85% of cases employed a sequential approach combining Angiojet with large-bore (≥10F) guiding catheters, forming the “PMT + large-lumen aspiration” paradigm. Approximately 20% of cases also utilized intermediate catheters for distal or residual thrombus. The rate of adjunctive CDT in Group C (14.3%) was comparable to that in Group B (14.3%), both higher than in Group A (0%).

### 3.4 Adjunctive venous angioplasty

**Figure 2:**
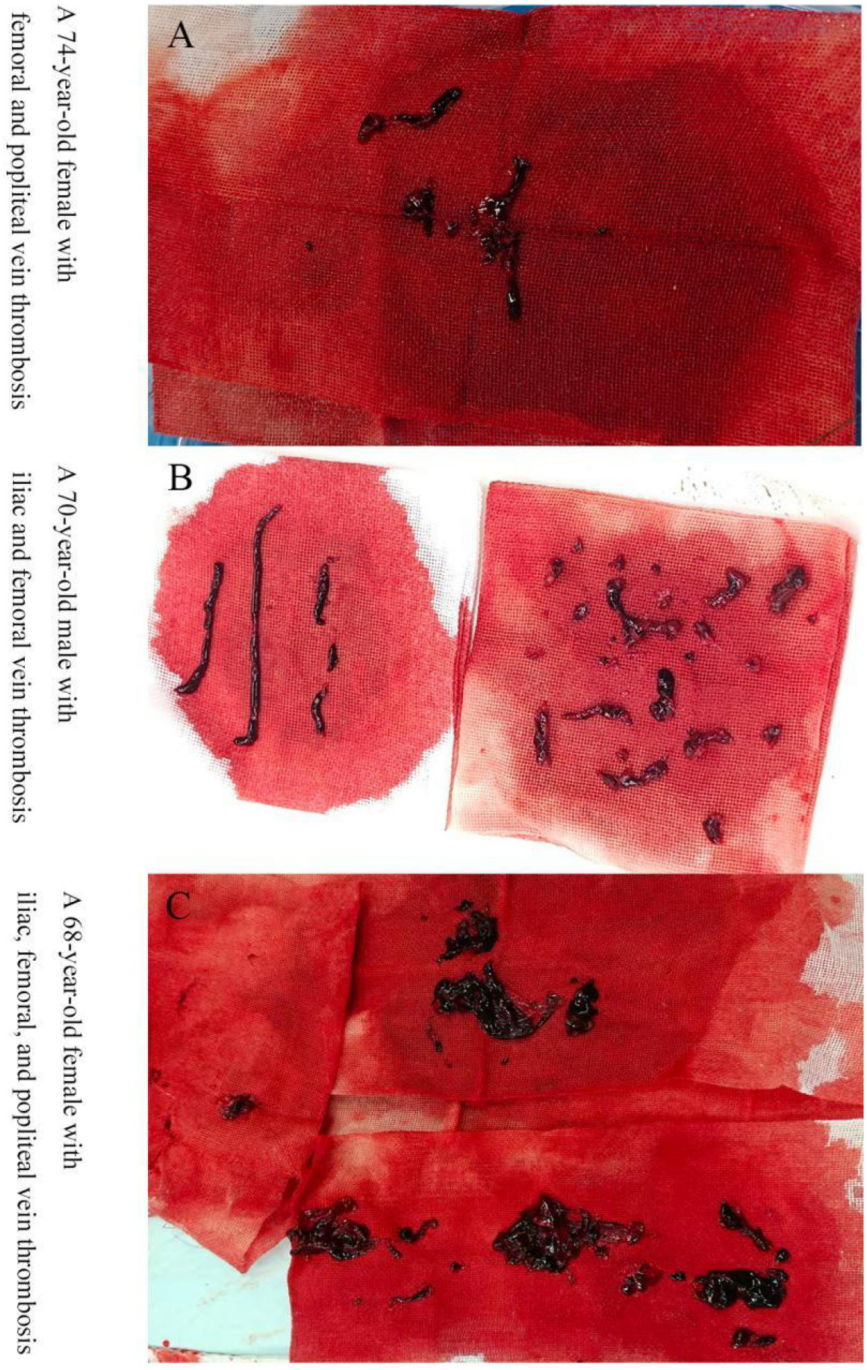
Thrombus extraction in three DVT cases treated with a combination of angiojet and adjunctive thrombectomy.

A gradient difference existed in the application of adjunctive venous angioplasty among the groups (Table 3). The balloon angioplasty rate increased from 54.5% in Group A, to 85.7% in Group B, to 96.0% in Group C (*P* < 0.001). The stent implantation rate showed a significant increasing trend across the three groups: 13.6% in Group A (all single stents), 28.6% in Group B (all single stents), and 58.9% in Group C (single or dual stents) (*P* < 0.001). The stents used were the Wallstent (Boston Scientific, USA) and the Zilver Vena venous stent (Cook Medical, USA), selected by the operator based on lesion length and vessel diameter. This indicates that the strategy in Group C aimed not only at thrombus removal but also leaned towards aggressive structural reconstruction of underlying venous outflow obstruction (e.g., iliac vein compression). Figure 2 illustrates the extracted thrombus material from three cases managed with the combined Angiojet and adjunctive thrombectomy approach, representing the intensive strategy (Group C) frequently applied to extensive or complex thrombosis as detailed in the results (Tables 1, 2).

**Table 3:** Comparison of primary treatment and adjunctive procedures.

Parameter	Overall (*N* = 232)	Group A (*n* = 22)	Group B (*n* = 35)	Group C (*n* = 175)	*P*-value
**Core Thrombectomy Device**					
Angiojet 8F Used, n/N (%)^a^	138/197 (70.1)	16/22 (72.7)	0/0 (-)	122/175 (69.7)	0.726
Angiojet 6F Used, n/N (%)^a^	59/197 (29.9)	6/22 (27.3)	0/0 (-)	53/175 (30.3)	0.775
**Adjunctive Procedures, *n* (%)**					
Balloon Angioplasty	210 (90.5)	12 (54.5)	30 (85.7)	168 (96.0)	**<0**.**001**
Stent Implantation	116 (50.0)	3 (13.6)	10 (28.6)	103 (58.9)	**<0**.**001**
Catheter-Directed Thrombolysis (CDT)	30 (12.9)	0 (0)	5 (14.3)	25 (14.3)	0.198

Bold *P*-value <0.05 indicates statistical significance.

a Denominator is the number of patients who received Angiojet in Groups A and C. Group B did not use Angiojet.

### 3.5 Perioperative parameters and procedural outcomes

Perioperative parameters correlated positively with treatment intensity (Table 4). The mean operative time was longest in Group C (134 ± 48 min), significantly longer than in Group B (115 ± 45 min) and Group A (98 ± 36 min) (*P* < 0.001). Intraoperative blood loss also showed a trend of Group C (66 ± 39 mL) > Group B (55 ± 35 mL) > Group A (44 ± 32 mL) (*P* = 0.008). The length of hospital stay was longest in Group B [5 (3–9) days], potentially related to older age and more comorbidities. Regarding descriptive outcome measures, Group B had the highest proportion of operative notes mentioning “partial thrombus residue” (22.9%), compared to 9.1% in Group A and 13.1% in Group C, though the intergroup difference was not statistically significant (*P* = 0.260).

**Table 4:** Perioperative outcomes and procedural characteristics.

Parameter	Overall (*N* = 232)	Group A (*n* = 22)	Group B (*n* = 35)	Group C (*n* = 175)	*P*-value
**Perioperative Metrics**					
Operative Time, min, mean ± SD	128 ± 48	98 ± 36	115 ± 45	134 ± 48	**<0**.**001**
Intraoperative Blood Loss, mL, mean ± SD	62 ± 38	44 ± 32	55 ± 35	66 ± 39	**0**.**008**
Hospital Stay, days, median (IQR)	4 (3, 7)	3 (2, 5)	5 (3, 9)	4 (3, 7)	**0**.**02**
“Partial Thrombus Residue” Documented	33 (14.2)	2 (9.1)	8 (22.9)	23 (13.1)	0.26

SD, Standard Deviation; IQR, Interquartile Range.

Bold *P*-value <0.05 indicates statistical significance.

## 4 Discussion

This study provides, through a systematic analysis of 232 endovascular DVT cases, the first clear depiction of the clinical landscape corresponding to three distinct treatment strategies based on the application of Angiojet (alone, combined, and its alternative). The key finding is that these three strategies are not mutually competitive but constitute a stratified and continuous spectrum of therapeutic decision-making, closely associated with the anatomical complexity of the thrombus and the patient's clinical condition. This finding deepens our understanding of individualized endovascular DVT treatment.

First, our data strongly confirm that the choice of treatment strategy primarily depends on the anatomical extent of the thrombus. Angiojet alone (Group A) and other devices alone (Group B) served primarily for limited (femoral/popliteal) thrombosis, aligning with the devices' design intent and economic efficiency principles (13). However, when the thrombus extends proximally to the iliac veins or even the inferior vena cava, clinical decisions almost uniformly steer towards the intensified strategy of Angiojet combined with other devices (Group C). This choice has a clear pathophysiological basis: extensive thrombus burden requires more potent clearance capability and a more flexible combination of tools. Angiojet can effectively loosen and fragment bulky thrombi (14, 15), while subsequent large-bore catheter aspiration can rapidly remove thrombus debris, prevent distal embolization, and improve efficiency (16). The finding that 22.3% of cases in Group C involved the IVC, with the vast majority managed by the combined strategy, is consistent with previous studies emphasizing the need for more aggressive intervention in proximal high-risk thrombosis (17, 18). DVT should not be viewed solely as a local vascular event but rather as a manifestation of a systemic prothrombotic state. As highlighted by Siniscalchi et al., hospitalized patients receiving non-invasive ventilation exhibit an increased risk of venous thromboembolism, underscoring the importance of recognizing acute clinical conditions as amplifiers of thrombotic risk (19). In our cohort, such systemic factors may have influenced both thrombus burden and treatment selection, particularly in Group B, where patients were older and had a higher prevalence of malignancy.

Second, different strategies resulted in distinct perioperative courses and treatment intensity. The significantly longer operative time and slightly greater blood loss in Group C should not be simplistically attributed to inherent inefficiency of the combined technique but rather understood as necessary resources expended for more thorough treatment of more complex disease (20). More importantly, the extremely high rates of balloon angioplasty (96.0%) and stent implantation (58.9%) in Group C reveal that the combined thrombus clearance strategy often serves as the prelude to a “one-stop” solution for venous obstruction. Figure 1 exemplifies this integrated approach, where thrombus removal is seamlessly followed by angioplasty and stenting in the depicted cases. In this cohort, stent implantation was primarily indicated for the management of underlying venous outflow obstruction unmasked after thrombus removal, most commonly iliac vein compression syndrome (May-Thurner anatomy), flow-limiting dissection, or significant residual stenosis (>50%) after balloon angioplasty. Stenting was also employed in cases of suboptimal angioplasty results. After thrombus removal, exposed underlying anatomical stenoses like iliac vein compression are addressed aggressively, which has been proven key to reducing PTS risk (21, 22). Therefore, the perioperative indicators of Group C reflect the overlapping of its dual roles of “diagnostic” and “therapeutic”.

Particularly noteworthy are the characteristics presented by Group B. Patients in this group were the oldest, likely had longer thrombus duration, and had a higher proportion of malignancy. This suggests that in clinical practice, for elderly patients, those in poorer general condition, or those with high bleeding risk factors (e.g., active malignancy), physicians may lean towards a seemingly more “gentle” and potentially more cost-controlled manual aspiration strategy (23). However, the trend towards the highest documented rate of “partial thrombus residue” in Group B (22.9%) raises an important question: Is the efficacy of manual aspiration alone sufficient for some thrombi, possibly the more subacute or chronic ones in this group. Although this trend did not reach statistical significance, it aligns with clinical intuition that tough thrombi may be more difficult to clear effectively without the aid of mechanical fragmentation (24). In contrast, Figure 2 demonstrates the substantial thrombus material that can be extracted using the combined approach, underscoring its potential for more complete clearance. This indirectly supports the potential value of PMT devices like Angiojet in improving the thoroughness of thrombus clearance and points the direction for future research comparing the long-term efficacy (e.g., patency rates, PTS incidence) of different strategies. Beyond mechanical thrombus removal, pharmacological agents may modulate clot structure and stability. Statins, for instance, have been shown to influence fibrin architecture and exert pleiotropic antithrombotic effects, potentially affecting thrombus resolution and recurrence risk (25). While our study focused on interventional strategies, adjunctive pharmacological modulation warrants further investigation as a complementary strategy to optimize outcomes. Although our study primarily reports perioperative and procedural outcomes, the ultimate goal of any DVT intervention is to improve clinically meaningful endpoints such as mortality, recurrence, and post-thrombotic syndrome (PTS). Large real-world registry studies have consistently demonstrated that statin use is associated with improved survival in patients with venous thromboembolism. Using data from the RIETE registry, Siniscalchi et al. showed that patients using statins at baseline had a significantly lower risk of all-cause death during anticoagulation for VTE compared to non-users (HR: 0.62; 95%CI: 0.48–0.79 after propensity score matching) (26). In a subsequent analysis focused specifically on acute pulmonary embolism, the same group reported that statin users had a 35% lower risk of 30-day all-cause mortality (OR: 0.65; 95%CI: 0.56–0.76) and a 58% lower risk of fatal PE (OR: 0.42; 95%CI: 0.28–0.62) (27). More recently, an analysis of 46,440 patients with isolated DVT demonstrated that statin users had a 23% lower risk of death at 3 months (aHR: 0.77; 95%CI: 0.69–0.86), with significant mortality reductions observed across proximal lower-limb DVT (aHR: 0.69; 95%CI: 0.50–0.95), distal lower-limb DVT (aHR: 0.48; 95%CI: 0.32–0.72), and upper-limb DVT (aHR: 0.81; 95%CI: 0.72–0.91) (28). Taken together, these findings suggest that integrating pharmacological risk modulation—specifically statin therapy—with interventional strategies may enhance long-term outcomes beyond the immediate procedural period.

Strengths of this study include the relatively large real-world sample size and detailed recording of surgical specifics, clearly revealing the internal logic of clinical decision-making. However, Several limitations should be acknowledged. First, the retrospective, non-randomized design introduces potential selection bias. Second, missing data (e.g., D-dimer dynamics, quantitative thrombus clearance scores) were handled by case-wise exclusion, which may affect generalizability. Third, the absence of long-term follow-up limits assessment of PTS and recurrence rates, which are critical for evaluating the true impact of different strategies. Fourth, the sample size of Group A (*n* = 22) and Group B (*n* = 35) is relatively small, which may limit the statistical power for subgroup comparisons (29).

Looking ahead, this study suggests that endovascular DVT treatment is moving towards a more refined “precision medicine” model. Future research should focus on developing predictive models integrating thrombus anatomical features, duration, biomarkers, and patient comorbidities to more objectively guide device selection, finding the optimal balance between “treatment efficacy,” “procedural risk,” “economic cost,” and “long-term outcome” (30, 31). Concurrently, there is an urgent need for well-designed prospective, randomized controlled trials to compare whether, for medium-burden thrombi, the combined strategy offers superior long-term venous patency and quality of life compared to single-device strategies (32).

## 5 Conclusion

In real-world clinical practice, endovascular treatment for lower extremity DVT has evolved into highly specialized stratified strategies. The use of Angiojet mechanical thrombectomy alone, its combination with other thrombus removal devices, or the use of other devices alone correspond to thrombotic disease spectra of varying complexity. The Angiojet combination strategy is foundational for managing extensive and complex DVT, with its longer operative time and higher rate of vascular interventions being a direct reflection of disease complexity and treatment thoroughness. The application of manual aspiration alone in specific populations also reveals the careful weighing of overall patient condition and treatment risks in clinical decision-making. This study provides important real-world evidence and a framework for consideration in the individualized endovascular treatment of DVT.

## Data Availability

The original contributions presented in the study are included in the article/Supplementary Material, further inquiries can be directed to the corresponding author/s.
